# Increasing disclosure of school-related gender-based violence: lessons from a systematic review of data collection methods and existing survey research

**DOI:** 10.1186/s12889-023-15526-w

**Published:** 2023-05-30

**Authors:** Clare Tanton, Amiya Bhatia, Jodie Pearlman, Karen Devries

**Affiliations:** London School of Hygiene and Tropical Medicine, 15-17 Tavistock Place, London, WC1H 9SH UK

**Keywords:** School-related gender-based violence, Children, Adolescents, Review, Mode of data collection, Survey

## Abstract

**Background:**

School-related gender-based violence (SRGBV) includes sexual, physical or psychological violence occurring in and around schools often perpetrated by teachers or peers. In this review, we focus on studies comparing how data collection methodologies affect children’s disclosures of SRGBV.

**Methods:**

We conducted a systematic review, searching nine databases for studies from high, middle and low-income countries using search terms related to violence, disclosure and data collection methodology. Records were initially screened by abstract and then full-texts were retrieved and data from eligible reports extracted. In this paper, we draw on results from this larger systematic review highlighting studies conducted with children which either collected data in schools or asked about violence in schools. We also describe methods compared and results of studies that were not conducted in schools, but that included children and young people. Finally, we describe how multi-country nationally representative surveys conducted in at least one low and middle-income country measure children’s experiences of SRGBV.

**Results:**

We screened 28,780 records, of which fourteen are included in this article. Only four studies compared data collection methodologies in schools or about violence in schools. These showed a 0 to more than 500-percent variation in the prevalence of violence measured using different data collection methodologies. An additional ten studies which were not conducted in schools, examined disclosure of violence in children and young people that was not specifically school-related. We assessed five multi-country national surveys that measured SRGBV. This limited evidence suggests that methods allowing increased anonymity (e.g. audio computer assisted self-interview, online surveys) may result in higher disclosure of violence, including SRGBV, than face-to-face interviewing. No studies included reported on safety, experiences of young people, or the costs of different methods. Multi-country national surveys used self-completion methods if completed in schools or face-to-face interviewing if completed in households, to measure SRGBV.

**Conclusion:**

Evidence on the impact of data collection method on SRGBV disclosure is limited, however current prevalence of SRGBV in international surveys used to monitor SDG progress may be underestimated due to data collection methods used. Further research on SRGBV should aim to test the effects of data collection methodology on the disclosure of violence. Efforts to improve the measurement of SRGBV is central to understanding the epidemiology, monitoring changes, and developing school and community-based programs as well as policies to prevent and respond to SRGBV.

## Background

School-related gender-based violence (SRGBV) “involves acts or threats of sexual, physical or psychological violence occurring in and around schools, perpetrated because of gender norms and stereotypes, and enforced by unequal power dynamics.” [1] Different acts of SRGBV can occur in an overlapping and interrelated fashion and may reinforce one another and interplay with other inequalities. Although there is controversy about how to define and measure the ‘gender-based’ element of SRGBV, estimates suggest that violence in schools may be more prevalent than violence at home. [2] Globally, about 60% of children aged 6–10 years report recent physical, and 60% report recent emotional, violence victimisation from peers at school. [2] Definitions of SRGBV also include corporal punishment due to the gendered nature of physical punishment practices [3]. Although robust data on prevalence of teacher violence are not routinely collected across countries, a recent systematic review reports that 46–95% of primary school students experience corporal punishment, [4] including in countries with legal prohibitions. Data on sexual harassment and sexual violence from children below 15 years old are also sparse, but 11% of students aged about 13–17 years across 96 countries report ‘being made fun of with sexual jokes, comments or gestures’. [5] There are no comparable national surveys with specific data on violence from teachers, although the Demographic and Health Surveys and the Violence Against Children Surveys include teachers as one possible perpetrator of sexual violence, and limited analyses of these data reveal that < 2% girls and < 1% boys report sexual violence from teachers [6–8]. The risk of school-related sexual violence victimisation is unequally distributed, with groups experiencing other inequalities at higher risk. In Uganda, for example, 20% of disabled, compared to 10% of non-disabled, primary school girls aged 11–14 years reported sexual violence victimisation, mainly from peers but also from teachers. [9] There are a myriad of potential consequences of SRGBV including outcomes related to physical health and health risk behaviours, poor mental health, perpetration of violence and poor educational outcomes. [10–13].

The Sustainable Development Goals (SDGs), a collection of 17 interlinked goals, the ‘blueprint to achieve a better and more sustainable future for all’, were set up by the United Nations General Assembly in 2015 and are intended to be achieved by 2030 [14, 15]. SRGBV is a barrier to realising key SDGs: to end abuse, exploitation, trafficking and all forms of violence against children in all settings (Target 16.2); to build and upgrade education facilities that are child, disability and gender sensitive and provide safe, non-violent, inclusive and effective learning environments for all (Target 4.a); and to achieve gender equality and reduce gender-based violence (Goal 5).

Collecting good quality data on SRGBV is important in order to understand the magnitude of the problem, to monitor progress towards the SDGs, and to design appropriate interventions. Currently, there are no routine international surveys that comprehensively ask about prevalence of different forms of physical, sexual and emotional violence from teachers, peers and others, or which systematically examine whether violence occurred within school environments. Most data on SRGBV comes from international survey datasets, which employ different definitions of violence, or ask about different perpetrators. [16–21] Other, more comprehensive, data on SRGBV come from smaller school-specific studies and cluster randomised trials which are testing school-based violence prevention interventions. [22–26] These data sources often have varying definitions of SRGBV and use different data collection strategies, all of which can yield very different prevalence estimates and make it extremely challenging to accurately estimate the overall prevalence of SRGBV, and to compare estimates across surveys or contexts.

In this review, we focus on studies comparing how data collection affects children’s disclosures of SRGBV in research. There is a growing body of research examining how data collection methods such as face-to-face interviewing, self-administered questionnaires, or list experiments, affect disclosure of intimate partner violence against adult women. [27–31] There is more limited research on how children respond to different collection methods, and how the choice affects the reporting of violence by children and young people. There are additional challenges around collecting data with children in school-based settings, with some key considerations being the age of the children and their cognitive capacity to engage with different modes of questionnaire administration and questions about violence; the presence of teachers and peers, who may have perpetrated the violence children are being asked to disclose; the nature of the physical location of data collection and ensuring privacy, which can be difficult in crowded classrooms; and navigating passive versus active parental consent and children’s rights around participation in research that may affect them.

In this paper, we aim to: (1) describe which methods have been tested in high, middle or low income countries to increase disclosure of experience and use of physical, sexual and emotional violence that is school-related or data that are collected within schools, commenting on ethical and safety aspects of methods; and (2) summarise other strategies that have been used with children and young people, which could increase disclosures of SRGBV, but that have not yet been tested in schools or in relation to SRGBV specifically. Finally, given the limited research on SRGBV in low- and middle-income countries (LMICs) and the reliance on studies from high-income countries, we (3) comment on the methods used in multi-country nationally representative surveys that measure violence against children and are conducted in at least one LMIC and assess if, and how, these surveys collect SRGBV information.

## Methods

In this paper, we draw on results from a larger systematic review (PROSPERO 2021 CRD42021235504) which examined studies that compared disclosures of violence with different data collection methodologies. Here we present data on the subset of studies where children were interviewed about school-related violence and draw on evidence from studies which have examined methods of collecting broader violence data from children. We also examine the data collection methods used in the main international multi-country surveys that measure violence against children and assess whether these surveys collect information on SRGBV including peer and teacher violence.

### Search strategy and screening

#### Review

For our larger systematic review, we searched eight databases (OvidSP Medline, OvidSP Embase, OvidSP PsycInfo, OvidSP Global Health, Ebsco CINAHL Plus, Clarivate Analytics Web of Science Core Collection, Wiley Cochrane Library, and World Health Organization Global Index Medicus) in February 2021. Search terms were developed to capture studies which compared disclosures of violence with different data collection methodologies. A search string was developed to include terms for: (1) violence (including terms related to and examples of acts of physical, sexual, emotional violence victimisation and perpetration, and adverse childhood experiences); (2) disclosure (including terms related to reporting, screening for violence and help-seeking); and, (3) data collection methodology (including terms related to comparative studies, trials, evaluations and experiment). Search strings were adapted for each database using advanced search syntax. We additionally hand searched the references of all identified systematic reviews for any additional articles. We did not search grey literature as the focus of this review was peer reviewed articles.

#### Multi-country surveys

To identify multi-country surveys that measure SRGBV, we conducted a narrative literature review and searched key organisational websites to find examples of surveys used to generate multi-country estimates of SRGBV. We selected a purposive sample of surveys to illustrate the types of data available to conduct multi-country analyses of SRGBV.

### Inclusion criteria

#### Review

For our larger review, all studies from high-, middle-, and low-income countries with (1) self-reported measures of violence (physical, sexual, emotional, homicide, bullying, or neglect perpetration or victimization) at any point in the life course, and (2) quantitative comparisons of data collection methods either within-individuals or between groups, among (3) children or adults were included. In this paper, we include two subsets of studies. For aim one, we include studies which were child-focused (aged 0–17 years) and did not include adults and either interviewed children in schools or asked about school-related violence as a proxy for those which may be most relevant for collection of data on SRGBV. For aim two, we expanded our scope to include studies which asked about other forms of violence that included both adults and children. Studies from any year, language or country were eligible for inclusion.

#### Multi-country surveys

Surveys were included if they measured a component of SRGBV and had been conducted in multiple countries including at least one LMIC based on the most recent World Bank definition of an LMIC [32].

### Data screening and extraction

#### Review

Screening was conducted by CT, AB and four other reviewers in two stages, using Covidence. [33] First, we screened the title and abstract of each article: 20% of articles were screened by two reviewers with an agreement rate over 99%. Two reviewers then screened each of the full texts of all articles that had not been eliminated. Disagreements were resolved by discussion among reviewers.

We extracted data on: study setting and population (age and sex); location of data collection; data collection methods compared; type of comparison (between or within individual); whether randomization was used; definitions of violence; and safety and quality characteristics. For each study, we extracted descriptive results and the results of statistical tests to compare data collection methods (e.g., adjusted odds ratios, kappa, sensitivity, specificity). CT, AB, and JP with support from four other reviewers extracted and checked the data: one reviewer extracted the data and a second reviewer checked each field. Discrepancies were resolved by discussion with a third reviewer if necessary. In two cases, the detail in the paper was insufficient, and the primary authors were contacted for further information.

To assess the measurement of SRGBV in the studies included in this paper, we extracted additional information from each paper along dimensions that may be important points of consideration for collection of data on SRGBV and in schools. This included whether: violence measures asked about specific behavioural acts; an element to capture the ‘gender-based’ nature of violence was included in the definition; the survey was anonymous for respondents; teachers were present during data collection; data were linked to individuals; the costs of administration and implementation logistics were reported; a child protection response framework was implemented; and, if feedback on the method of data collection was sought from participants.

#### Multi-country surveys

We extracted information about the survey program, mode of administration, location of interview, study population, violence measures, whether teacher and/or peer violence was measured, efforts to measure safety or privacy; and evidence of a child protection response plan.

### Analysis

#### Review

We first identified studies from our broader systematic review which either interviewed children in schools or asked about school-related violence. We describe characteristics of these included studies, and narratively synthesise results according to our aims. To address our first aim, we synthesised information about (a) study design and methods, (b) modes of administration, (c) the safety and ethics of each study and then assessed gaps in knowledge, (d) study findings and any sub group analyses by age and sex. We did not attempt to quantitatively assess publication bias as there were too few studies reporting on any specific outcome to make this possible. Instead, we used the Joanna Briggs quality checklist relevant to the particular study design to determine whether studies met sufficient quality standards to be included in the review [34–36]. To address our second aim, we then described the methods compared and results of studies that asked about other forms of violence and included children and young people alongside adults.

#### Multi-country surveys

To address our final aim, we described methods used in nationally representative surveys that measure violence against children.

## Results

### What methods have been tested to increase children’s disclosure of SRGBV?

**Fig. 1:**
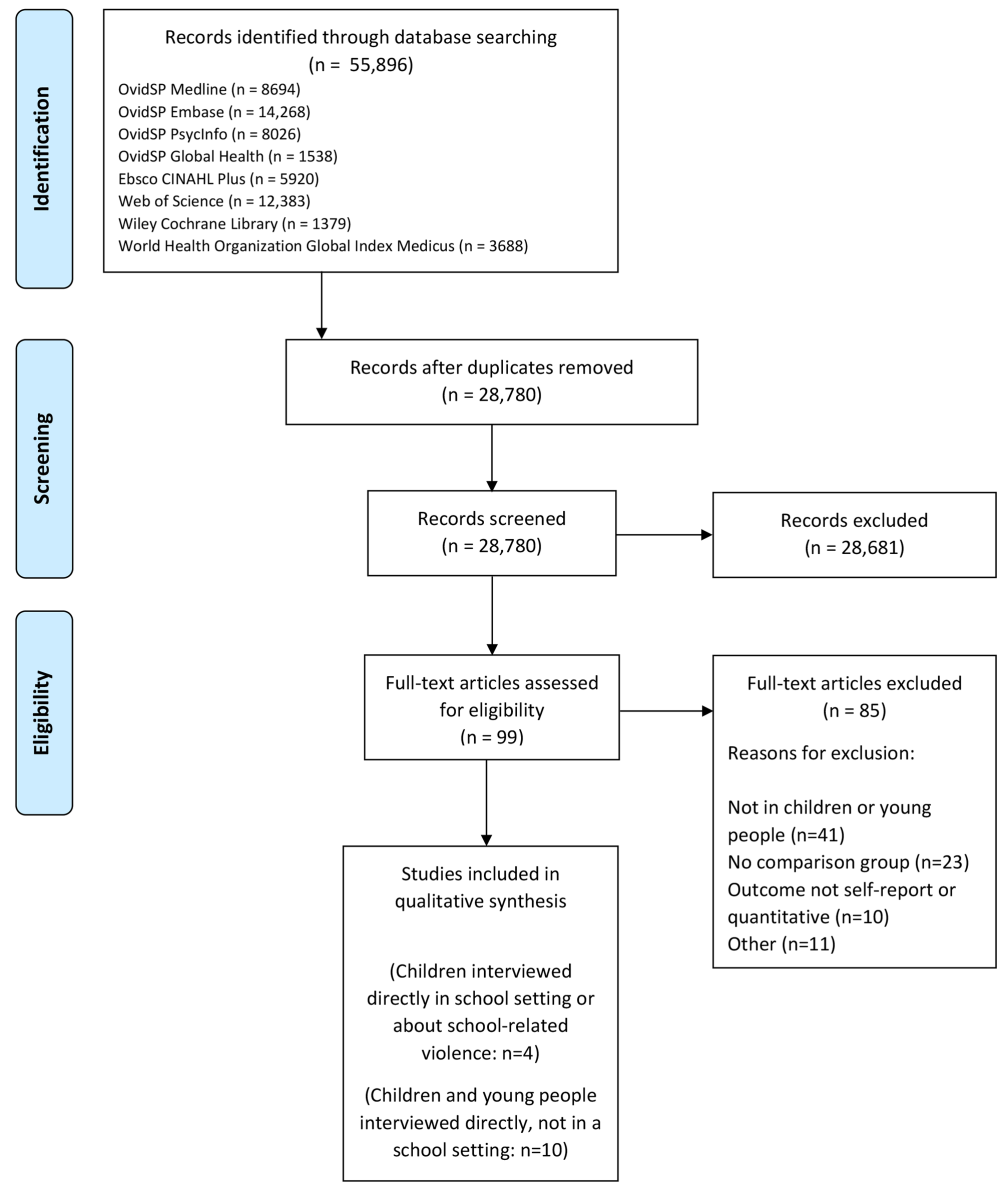
Prisma flow diagram [37]

Titles and abstracts were screened for 28,780 records. The full text of 99 articles was assessed, and 55 studies were included in our larger review. Only 4 studies met our inclusion criteria for this paper (Fig. 1). All studies scored sufficiently highly in the quality control checklist to be included.

Four studies interviewed children aged between 8 and 17 years either in a school setting, or about school-based violence (Table 1). All studies sampled from schools or communities, and were not nationally representative. One study was conducted in each of the USA, Finland, Canada and Uganda. [38–41] Three of the four studies were conducted in schools and one asked about school violence in a research office setting. Studies measured a range of physical, sexual and emotional violence types. All looked at violence victimisation with one also examining violence perpetration. Three of four studies included some act-based measures of violence, while in the fourth study [41] it was unclear what questions had been asked. No studies attempted to quantify whether motivations for violence were gender-based, instead measuring acts of violence only. Two studies used a randomized design to assign participants to different modes of data collection method on violence reporting. Most studies used a between-individuals design, comparing two different sets of individuals assigned to receive different data collection methods. [39–41] One study used a within-individuals design and compared the same individuals’ responses to different data collection methods. [38].

**Table 1:** Design characteristics of included studies

Authors	Country	Participant age; recruitment location; data collection location	Violence outcomes	Gender-based included in definition	Randomized experiment	Sample size	Between or within individual comparison*
1) Barr, 2017 [38]	Uganda	11–14 years, primary schools; primary schools	Victimisation Forced sex from any perpetrator (lifetime)	No	No	3843	Within individuals
2) Bethel, 2016 [39]	United States	8–12 years, database of families signed up to participate in various research projects; office	Victimisation Bullying by other students (past month)	No	No	60	Between individuals
3) Hilton, 2003 [40]	Canada	Grade 11 students (aged 16–17 years); secondary schools; secondary schools	Victimisation IPV (physical, emotional, sexual) (past year) Perpetration IPV (physical, emotional, sexual) (past year)	No	Yes	410	Between individuals
4) Kivivuori, 2013 [41]	Finland	Grade 9 students (aged 15–16 years); secondary schools; secondary schools	Perpetration Physical violence (lifetime and past year), Bullying (ever and past 12 months)	No	Yes	924	Between individuals

*Between individual design compared two different sets of individuals assigned to receive different data collection methods. Within-individuals design compared the same individuals’ responses to different data collection methods.

Table 2 describes the location of data collection, and how each mode of administration was operationalised. Three of the four studies collected data in schools; [38, 40, 41] one asked students about their experience of bullying, but was administered in an office setting. [39] Two studies compared face-to-face interviews to other methods. [38, 39] In these studies, face-to-face interviews were conducted by a researcher or a robot. Modes of administration that were not face-to-face included: (1) Sealed Envelope Method where children responded on a piece of paper and placed it in a sealed envelope and put that into a box in the classroom; [12] (2) a paper self-report questionnaire; [40] (3) a scenario-based report alongside a self-report questionnaire, where participants first listened to an audiotape of actors narrating and performing specific violent acts and were then asked to complete a questionnaire asking about victimisation or perpetration of these acts; [40] and (4) a web-based survey which children completed in school. [41] Most modes of administration used required a researcher to be present. Hilton and colleagues had teachers present for both modes of administration, [40] while Kivivuori and colleagues tested whether the presence of a teacher affected disclosure. [41] Although all studies described the implementation logistics, no study reported the costs of administration.

**Table 2:** Modes of administration and logistics of included studies

	Location of data collection	Mode of administration	Interviewer	Mode of response	Who is present during administration	Costs of administration reported	Implementation logistics reported
1) Barr, 2017 [38]	School	1. Face-to-face interview (FTFI),	Researcher	Oral	Researcher	No	Yes
		2. Sealed Envelope Method (SEM)	None	Picture/written	Researcher supervising		
2) Bethel, 2016 [39]	Office	1. Face-to-face interview (FTFI) by robot,	Robot	Oral	Robot	No	Yes
		2. Face-to-face interview (FTFI) by human interviewer	Researcher	Oral	Researcher		
3) Hilton, 2003 [40]	School	1. Standard-method self-report questionnaire,	None	Written	Researcher and teacher*	No	Yes
		2. Scenario-based report with self-report questionnaire	None	Written	Researcher and teacher*		
4) Kivivuori, 2013	School	1. Online self-report (supervised by respondent’s teacher),	None	Web-based	Teachers	No	Yes
		2. Online self-report (supervised by an external research assistant)	None	Web-based	Researcher		

*Paper not very clear as to who is present

Table 3 shows the safety characteristics of included studies. Child participation in measure development and testing was minimal, with only one study seeking children’s feedback about the methods used. [39] The design of all four studies allowed for anonymous disclosures at the point of data collection; one study allowed response to be linked to individuals and for child protection responses to be implemented in response to disclosures. [38] No study asked questions to participants or specifically assessed aspects related to the safety of respondents during data collection.

**Table 3:** Safety of included studies

Authors	Ethical approval described	Consent	Feedback from participants sought	Anonymous for respondents	Safety assessed	Data linked to individuals	Child protection response framework implemented	
1) Barr, 2017 [38]	Yes	Headteacher consent, Parental information; child consent	No	Yes	No	Yes	Yes	
2) Bethel, 2016 [39]	Yes	Parental consent, child assent	Yes	Yes	No	No^a^	No	
3) Hilton, 2003 [40]	None reported	Parental information, child consent	No	Yes	No	No^a^	No	
4) Kivivuori, 2013 [41]	None reported	Not reported	No	Yes	No	No^a^	No	

^a^in these studies, linkage was not implemented, although the methods do not preclude that

Table 4 shows the levels of disclosure for the different data collection methods used in each study. The differences between levels of disclosure by the data collection methods tested ranged from a negligible 6% to a highly significant > 500% across studies. [38, 41] Too few studies met the inclusion criteria to comment definitely on differences in methodological approaches, but in two studies, on sexual violence and bullying respectively, there was a suggestion that more anonymous methods (a sealed envelope, [38] and a robot, [39] both compared to a face-to-face interview) were associated with higher levels of disclosure. One study examining whether teachers versus external research assistants supervising online self-reporting of physical violence and bullying perpetration affected disclosure found no evidence of a difference in levels of disclosure. [41] The final study compared a standard self-reported questionnaire to a ‘scenario-based measure’ alongside a self-reported questionnaire. [40] Both were administered in the presence of teachers and/or trained research assistants. This study found that the self-reported questionnaire yielded higher levels of disclosure of both physical violence victimisation and emotional violence victimisation and perpetration. Reports of sexual violence were higher with scenario-based report. [40] All studies analysed results separately by sex, which is important given the gendered nature of both violence and disclosure, but only one study analysed results separately by age. [38] In terms of analysis by sex, in one study there was also a suggestion that this gap was gendered with sealed envelope increasing disclosure of sexual violence more in boys than girls. [38] In another study there was some suggestion that reports from female students were higher with external researchers than teachers whereas for males there was no difference. [41] The other studies either did not find a difference by sex, [40] or did not have sufficient endorsements of violence to compare by sex. [39].

**Table 4:** Results of studies

Authors	Type of Violence	Method and prevalence of violence	Comparison	Subgroup analyses: by age	Subgroup analyses: by sex	Interpretation
1) Barr, 2017 [38]	Forced sex (lifetime)	Estimates: **Victimisation** 1. Face-to-face interview (FTFI): 1.1% [REF] 2. Sealed envelope method (SEM): 7.0%	Comparison: **Victimisation** 1. FTFI sensitivity: 13.1% (95% CI 9.3–17%) 2. FTFI specificity: 99.8% (95% CI 99.6–99.9%) 3. Positive likelihood ratio: 66.7 (95% CI 29.9–149.0) 4. Negative likelihood ratio: 0.87 (95% CI 0.83–0.91)	Yes	Yes	**Victimisation** *Disclosure higher with sealed envelope method compared to face-to-face interview. Strong evidence* Subgroup analyses showed boys less likely than girls to disclose in FTFI but no difference by sex when using SEM. No differences in disclosure by age.
2) Bethel, 2016 [39]	Bullying by other students (past month)	Estimates: **Victimisation** 1. Robot administered face-to-face interview (FTFI): 11.7% [REF] 2. Human administered FTFI: 3.3%	Comparison: **Victimisation** 1. Chi-squared (1, 60): 6.67, p-value = 0.071	No	No	**Victimisation** *Disclosure higher with robots compared to face-to-face interview. Weak evidence.*
3) Hilton, 2003 [40]	IPV perpetration and victimisation (physical, emotional, sexual) (past year)	Estimates: **Victimisation** Physical 1. Self-report on questionnaire: 58.0% [REF] 2. Scenario-based report: 34.0% Emotional and sexual violence victimisation only presented in graphs **Perpetration** Physical 1. Self-report on questionnaire: 65.0% [REF] 2. Scenario-based report: 25.0% Emotional and sexual violence perpetration only presented in graphs.	Comparison: **Victimisation**: Physical 1. Kappa: 0.31, p-value < 0.001 Emotional 1. Kappa: 0.28, p-value < 0.01 Sexual 1. Kappa: 0.41, p-value < 0.001 **Perpetration** Physical 1. Kappa: 0.11 (nonsignificant) Emotional 1. Kappa: 0.12, p < .05 Sexual 1. Kappa: 0.51, p-value < 0.001	No	Yes	**Victimisation** *Disclosure higher with self-report on questionnaire compared to scenario-based report for physical and emotional violence victimisation. Strong evidence.* *Disclosure higher with scenario-based report compared to self-report on questionnaire for sexual violence victimisation. Strong evidence.* Subgroup analyses showed no difference by sex on the effect of method on disclosure for physical, emotional or sexual violence victimisation. *Significant concordance between methods for victimisation (physical, emotional). Strong evidence.* **Perpetration** *Significant concordance between methods for perpetration (emotional and sexual violence). Strong evidence.* *No significant concordance between methods for perpetration of physical violence* Subgroup analyses for physical or emotional violence perpetration showed few differences by sex in self-report on questionnaire, but higher disclosure among boys than girls in scenario-based report. Subgroup analyses for sexual violence perpetration showed disclosure higher among boys than girls in self-report questionnaire but not scenario-based report.
4) Kivivuori, 2013 [41]	Physical violence (lifetime and past year)	Estimates: **Perpetration** Lifetime 1. External researcher: 17.5% [REF] 2. Teacher: 19.1% Past year 1. External researcher: 6.6% [REF] 2. Teacher: 6.2%	Comparison: **Perpetration** Lifetime 1. Chi-squared (df = 1): 0.52, p-value not significant Past year 1. Chi-squared (df = 1): 0.08, p-value not significant	No	Yes	**Perpetration** *Disclosure higher with teachers compared to external researchers for lifetime physical violence but not past year violence. Weak evidence.*
	Bullying (ever and past 12 months)	Estimates: **Perpetration** Lifetime 1. External researcher: 50.5% [REF] 2. Teacher: 46.6% Past year 1. External researcher: 18.1% [REF] 2. Teacher: 19.6%	Comparison: **Perpetration** Lifetime 1. Chi-squared (df = 1): 1.44, p-value not significant Past year 1. Chi-squared (df = 1): 0.23, p-value not significant 2. Cramer’s V: 0.18	No	Yes	**Perpetration** *Disclosure higher with external researchers compared to teachers for lifetime bullying but not past year bullying. Weak evidence.* Subgroup analyses showed few differences by sex on the effect of method on disclosure.

### Other data collection methods to increase disclosures of violence against children

We now turn to our second aim, to describe other data collection methods for improving the disclosure of violence in research with children that have not been tested in schools or in relation to SRGBV. Drawing on our larger review, we found ten additional studies that included children and young people which were not conducted in schools or about SRGBV, but could be relevant for SRGBV research. Seven of these studies were conducted in America, [27, 42–47] one in Kenya, [48] Australia, [49] and Israel. [50] Most data were collected in health facilities. Five of these studies measured violence victimization, two studies measured perpetration [44, 47] and three studies measured both victimisation and perpetration. [43, 45, 46] Eight of the ten studies included a comparison between a form of face-to-face interviewing and other methods. Here data were mixed on whether more anonymous methods resulted in higher reporting. One study found no difference in disclosure of either perpetration or victimisation of physical or sexual violence between face-to-face interviewing, telephone interview, written questionnaire and automated telephonic data collection system. [45] In contrast, two studies found that more anonymous methods – a web survey and audio computer-assisted self-interview (ACASI) – were associated with increased reports of sexual violence compared to face-to-face interview. [48, 49].

In two other studies, both administered in healthcare settings, face-to-face interviews with individuals that young people may trust found higher levels of disclosure than written methods. In one study which measured physical violence in childhood with adolescents and young adults attending a youth friendly, primary care clinic, in-person, unstructured screening yielded significantly higher odds of disclosure compared to written screening. [27] In the other study, face-to-face interviewing by a nurse yielded a significantly higher proportion of disclosures compared to a written questionnaire for experiences of physical and sexual intimate partner violence (IPV). [42].

Two studies looked at methods of augmenting face-to-face interviewing. In one, when face-to-face interviews were augmented with drawing, higher disclosure was found compared to a face-to-face interview without drawing. [50] In another, a weekly face-to-face interview resulted in higher reporting compared to an interview conducted using a life events calendar for any violence. [44].

Both studies that did not test face-to-face interviewing as a mode of administration compared written self-administered questionnaire to other modes of administration. One study found ACASI was associated with higher disclosure for both victimization and perpetration. [46] A final study, among juvenile sex offenders, found a polygraph was associated with higher reporting of bestiality. [47].

Drawing on the findings from studies testing methods in relation to SRGBV, as well as the studies testing methods to ask children about violence, Table 5 outlines strategies to increase disclosures in SRGBV data collection used in these studies. For those methods that may be scalable, we consider potential ease of implementation, privacy and confidentiality issues, and whether child protection responses could be initiated. We have not included polygraph in this table since we do not consider this either an ethical or practical form of data collection. [47] ACASI is the method for which there is most evidence for higher disclosure compared to face-to-face interviewing, and computer-assisted self-interview (CASI) could be an alternative to ACASI where levels of literacy are higher. Both methods increase anonymity. Both methods generally require a shorter questionnaire length, particularly for ACASI, as well as some degree of computer literacy and, for CASI, good participant reading skills. Although these methods are likely to be cheaper to implement than face-to-face interviewing, they require a degree of privacy so that devices cannot be overseen. This may be challenging to achieve if classrooms are crowded and especially if data collection is overseen by teachers, and this may impact on reporting. ACASI and CASI also require consideration of the potential for participant distress on responding to the questions if data collection is carried out in a classroom setting and since interviewers may not be present to make immediate referrals. Written questionnaires have the same challenges, and also require a simpler questionnaire since complex skip patterns cannot be built in. They also tend to have lower quality data than computer options. For data collection not occurring in the school setting, other options include telephone interviewing and online surveys for which evidence is unclear as to the likely effect on disclosure. Telephone interviews are more time limited than face-to-face interviewing and online surveys have the same limitations as CASI interviews, with the additional challenge of internet connectivity. For remote methods, researchers are also unable to ensure the privacy of the data collection. There are other methods such as sealed envelopes that can be used to augment other modes of data collection for particularly sensitive questions. Existing studies would suggest that such methods are likely to result in increased disclosure, and this is likely to be true of particularly sensitive questions.

**Table 5:** Summary of promising methods to collect SRGBV data

Method	Studies testing methods to improve disclosure in children and young people	Ease of implementation	Privacy and confidentiality, deductive disclosure	Child Protection Responses; other ethical considerations	Implication for disclosures of violence
**Interviewer administered methods**					
FTFI	Diaz, 2017 [27] McFarlane, 1991 [42] Roberts, 2005 [44] Reddy, 2006 [43] Rosenbaum, 2006 [45] Hewett, 2004 [48] Bradford, 2015 [49] Katz, 2010 [50] Barr, 2017* [38] Bethel, 2016* [39] Hilton, 2003* [40]	Easy to administer; more costly than self-completion methods	Can maintain privacy depending on interview location; if multiple follow up questions are asked, potential for deductive disclosure	Yes; able to provide support to distressed participants	Likely to be lower, especially for more sensitive questions
FTFI with drawing	Katz, 2010 [50]	Harder to administer and to scale up; may be good for younger children	Can maintain privacy depending on interview location	Yes; able to provide support to distressed participants	Possibly higher
FTFI interview with Robot	Bethel, 2016* [39]	Challenging to administer	Can maintain privacy depending on interview location; if multiple follow up questions are asked, potential for deductive disclosure	No, unless recorded and listened to immediately; able to provide support to distressed participants	Unclear
Telephone interview	Reddy, 2006 [43] Rosenbaum, 2006 [45]	Easy to administer; more limits to questionnaire length than face-to-face; requires access to telephone	Participant has to ensure privacy	Yes, if responses are linked to individual identifying information; more difficult for interviewer to support participant if distressed	Unclear
**Self-completion methods**					
CASI	No studies compared this method but it is a computer version of written questionnaire and in-person version of online survey	Easy to administer; can build in routing so better quality data; dependent on good reading skills among participants; dependent on some computer literacy	Can maintain privacy if device is not overlooked; so may be difficult to implement in crowded classrooms; if multiple follow up questions are asked, potential for deductive disclosure	Yes, if responses are linked to individual identifying information; may not be desirable to implement in crowded settings due to possibility of participant distress	Likely to be higher in most settings because method allows privacy
ACASI	Diaz, 2017 [27] Hewett, 2004 [48] Turner, 1998 [46]	Easy to administer; can build in routing so better quality data; requires limited questionnaire length; dependent on some computer literacy	Can maintain privacy if headphones are used and device is not overlooked; so may be difficult to implement in crowded classrooms; if multiple follow up questions are asked, potential for deductive disclosure	Yes, if responses are linked to individual identifying information; may not be desirable to implement in crowded settings due to possibility of participant distress	Likely to be higher in most settings because method allows privacy
Online survey	Bradford, 2015 [49] Kivivuori, 2013* [41]	Easy to administer; can build in routing so better quality data; dependent on good reading skills among participants; requires limited questionnaire length; requires internet connectivity	Participant has to ensure privacy; requires adequate online security	Yes, if responses are linked to individual identifying information	Likely to be higher in most settings because method allows privacy If method is supervised by a teacher or researcher, this may affect disclosure
Written questionnaire	Diaz, 2017 [27] McFarlane, 1991 [42] Reddy, 2006 [43] Rosenbaum, 2006 [45] Turner, 1998 [46]	Easy to administer; requires simpler questionnaire as routing can’t be built in; dependent on good reading skills among participants	Location of data collection can determine privacy	Yes, if responses are linked to individual identifying information	Likely to be higher in most settings if method allows privacy
Automated telephone data collection (ATDC) system	Reddy, 2006 [43] Rosenbaum, 2006 [45]	Easy to administer; does not depend on literacy	Participant has to ensure privacy	Yes, if responses are linked to individual identifying information	Likely to be higher in most settings if method allows privacy
Sealed envelope	Barr, 2017* [38]	Easy to administer	Privacy and confidentiality ensured	No	Likely to be higher in most settings because method ensures anonymity
**Other methods**					
Life events calendar	Roberts, 2005 [44]	Challenging to administer	Can maintain privacy depending on interview location and whether it is self-complete or used within a face-to-face interview May help with recall	Yes	Unclear
Scenario based report	Hilton, 2003* [40]	Challenging to administer	Can maintain privacy depending on interview location and whether it is self-complete or used within a face-to-face interview	Yes, able to provide support to distressed participants if within a face-to-face interview; or if responses are linked to individual identifying information if self-complete	Unclear

* Indicates studies included in this paper which interviewed children in schools or asked about school-related violence

### Summary of practice in large-scale violence surveys

Finally, we turn to a discussion of current practice in large-scale, nationally representative surveys that collect information on SRGBV and are routinely administered in at least one LMIC (Table 6). We are aware of five such surveys. Three surveys are conducted in schools and are all self-administered: Global School Health Surveys (GSHS), Health Behaviour in School-Aged Children Surveys (HBSC), and Trends in International Mathematics and Science Study (TIMSS) & Progress in International Reading Literacy Study (PIRLS) using paper and in some surveys additionally computers/tablets. [17–19, 21] The remaining surveys are interviewer-administered, face-to-face surveys conducted in households. Four out of five surveys are conducted among children and youth and include girls and boys. In contrast, the Demographic and Health Surveys (DHS) are conducted among women of reproductive age and only include 15–18-year-olds. Four surveys measure physical violence from peers; two measure emotional violence from peers, and two include measures of sexual violence from peers. Although the GSHS measures multiple types of violence, the HBSC and TIMSS & PIRLS [17, 19, 21] measure multiple types of violence and ask specifically about peers as perpetrators. Only two surveys (Violence Against Children and Youth Surveys (VACS) and DHS) include a measure of teachers’ physical, sexual or emotional violence. [16, 20] However, both surveys first ask whether respondents have experienced specific behavioural acts of violence, and then linked to these acts, allow respondents to select teachers as one of a list of perpetrators of violence. [16, 20] Based on our experience, this type of questioning may lead to lower prevalence estimates compared to asking respondents directly if a specific perpetrator has used a range of behavioural acts of violence against them. The DHS measured interruptions, and the VACS was the only survey to seek feedback from participants.

**Table 6:** Selected multi-country surveys with at least some LMIC representation with SRGBV information

Survey name	Description	Mode of administration and location	Population for violence questions	Violence measures (type and time frame)	Teacher violence	Peer violence	Measures safety and privacy	Violence/Child Protection response plan included in questionnaire
Global School Health Surveys (GSHS)* Round 3 (E.g. Tanzania, 2015) [18]	109 countries in all regions except Europe and North America (mainly LMICs) Data on health behaviours and protective factors among students	Self-administered (paper via computer-scannable answer sheet) Classroom	Students (13–17 years)	Any violence, past 30 days Physical violence, past year Bullying (physical, sexual, emotional), past 30 days	Not measured	Not measured	No	No
Violence Against Children and Youth Surveys (VACS) (E.g. Zimbabwe, 2017) [16]	25 countries (all LMICs) Measures prevalence, nature, and consequences of violence against children	FTFI Household	Males and females (13–24 years)	Physical violence (includes IPV), past year and lifetime Threat of physical violence, past year and lifetime Sexual violence, past year and lifetime Emotional violence, past year and lifetime	Teachers included as perpetrators of physical, sexual, emotional violence	Module on physical violence by peers; peers included as perpetrators of physical and sexual violence and threat of physical violence	Yes – asks for feedback on participating in the survey Interviewers trained to respond appropriately to interview interruption, e.g. by rescheduling, moving to a more private location, switching to a non-sensitive mock questionnaire	Yes
Health Behaviour in School-Aged Children Surveys (HBSC) (E.g. 2013/14)^†[21]^	50 countries across Europe and North America (mainly high-income settings with some LMICs) Measures young people’s well-being, health behaviours and their social context; conducted every four years	Self-administered (can be managed by researchers or school staff; paper or electronic) Classroom	Young people attending school aged 11, 13 and 15	Physical violence, past year Bullying (bullying others and been bullied; physical, emotional), past couple of months	Not measured	Module on bullying refers to bullying at school	No	No
Trends in International Mathematics and Science Study (TIMSS) & Progress in International Reading Literacy Study (PIRLS) (E.g. TIMSS 2019) [17] (E.g. PIRLS 2016) [19]	64 (TIMSS) / 50 (PIRLS) countries (mainly high-income settings) Assesses the home, community, school and student factors associated with student achievement in mathematics and science at the fourth and eighth grades	Self-administered (paper or computer/tablet) Classroom (student questionnaire); online (teacher, school and home questionnaires)	Students enrolled in the fourth (TIMSS & PIRLS) and eighth grades (TIMSS), and their parents, teachers and school principals	Bullying (physical, emotional), during this year	Not measured	Peer physical violence included in module on School Discipline and Safety in the School Questionnaire Peer physical and emotional violence in Grades 4 and 8 Student Questionnaire	No	No
Demographic and Health Surveys (DHS) Round 7 (E.g. Pakistan, 2017-18) [20]	> 90 countries (mainly LMICs) Collects data on population, health and nutrition, with a focus on women of reproductive age. Module 17 is on domestic violence	FTFI Household	Women of reproductive age (15–49 years) ^‡^	Physical violence (including during pregnancy), since aged 15 and past year Sexual violence, past year and lifetime IPV (physical, sexual, emotional, and combination of types), past year and lifetime	Teachers included as perpetrators of physical and sexual violence	Own friend/acquaintance included as perpetrators of physical and sexual violence	Yes – interviewers interrupt or terminate domestic violence interview if privacy is breached	Respondents provided with information and referrals for services available for those experiencing domestic violence or in need of services

*Information refers to the Core questionnaire. Core Expanded questions are also available which contain additional violence-related questions

^†^An updated 2017/18 protocol is also available which requires registration

^‡^ Women are the focus of the DHS-7, but any knowledgeable person aged 15 or older living in the household responds to the Household and Biomarker Questionnaires and men of reproductive age respond to the Man’s Questionnaire

Note: These selected surveys which have information about SRGBV, including in LMIC contexts and do not represent all data sources on SRGBV globally.

## Discussion

This review brings together existing evidence on methodological considerations around SRGBV data collection. Our first aim was to describe approaches used to improve the disclosure of violence in the context of studies that interviewed children in schools or about school-related violence. We found only four studies testing ways to support increased disclosures from children on SRGBV, [38–41] and only seven different methods tested. Despite the limited evidence, we found a large range in the ‘prevalence gap’ generated by different methods. Despite having only four studies, the ‘prevalence gap’ between different methods which have been tested of relevance to SRGBV was up to a > 500% increase in prevalence. In one study, anonymous methods also increased disclosures more for boys, and had the effect of equating the prevalence of forced sex in boys and girls. If this finding was replicated in other studies it would have enormous implications for our targeting of interventions and for violence research in general. Only one other study found a gender difference [41] in the difference between method of data collection with reports of physical violence and bullying perpetration higher from female students when data collection was supervised by external researchers than teachers whereas for males there was no difference. It may be that more anonymous methods are important for increasing disclosures to more sensitive questions, and this may differ by sex. There were insufficient studies to comment on whether methods increased disclosure differently by age or type of violence.

Current evidence on methods to increase disclosure of SRGBV is severely limited with no age overlap between studies, only one study from a low-income country and few estimates of violence overall. From the four key studies included, it seems unlikely that robots will become a widely used data collection tool. The scenario-based method involving actors also presents obvious difficulties around the training of actors, designing and validating the audiotape and, most important, the accurate portrayal of specific forms of violence; hence in our view this method is likely to be challenging to design for small-scale studies, and unlikely to be useful in large-scale survey data collection. The sealed envelope method is low-tech and easy to implement, so could be considered for use at scale. However, given it is anonymous, individual-level child protection follow up becomes challenging, as does linking data gathered to other sociodemographic variables. From an ethical and safety perceptive, it also seems unadvisable to have teachers (who may be perpetrators of violence themselves) present during survey data collection on that topic, despite Kivivuori’s findings of no difference. [41] We note that in Finland, where Kivivuori’s study [41] took place, physical violence from teachers is likely to be extremely low prevalence, and it is likely that in other settings where prevalence of teacher violence is higher, different effects on disclosure might be found if the experiment was replicated. We did not find any studies conducted in relation to SRGBV examining CASI or ACASI, using video or telephone interviewing, or mailed questionnaires, list experiments or other methods.

Only one study asked about children’s views of different methods that were being compared. [39] Few studies reported on ethical and safety considerations related to different methods, and several studies appeared to administer questions in contexts where privacy could have been difficult to maintain, for example, in the presence of teachers and/or peers who could have perpetrated the violence children were being asked to disclose. It is important to get information on how children feel about these different methods because of the potential for re-traumatisation with such sensitive questions. Data are mixed on the impact of violence questions on participants. Some research found that one in four participants were upset by survey violence questions, and those upset were more likely to be younger, [51] however in a qualitative follow-up with primary school children participating in a violence survey, disclosure was found to generally be a positive experience with children not finding the interview traumatic. [52].

Our second aim related to other methods from research with children on violence that could be used in the context of SRGBV research. Although several other approaches emerged (e.g., drawing, ACASI, online surveys, phone interviews), firm conclusions about which methods increase SRGBV disclosure cannot be drawn. However, there is some suggestion that anonymous methods will result in higher levels of disclosure. Evidence from studies conducted on reporting of other sensitive behaviour supports this. A review of the effects of questionnaire delivery mode on the reporting of sexual behaviour included 26 studies and found that ACASI and CASI increased disclosure. [53] A study in Malawi found that young people were more likely to report having sex with a teacher or a relative in ACASI than a face-to-face interview. [54].

With regard to our third aim, we find that several widely-implemented school-based surveys in LMICs use self-completion methods, which provide anonymity but have limitations in low literacy settings. In contrast, international household surveys which ask about SRGBV use face-to-face interviewing which is more accessible to participants but also reduces anonymity. All of these limitations imply that current prevalence of SRGBV in international surveys used to monitor SDG progress is underestimated, and that efforts to measure SRGBV in smaller scale studies or cluster randomised trials may also be affected by reporting biases due to mode of administration.

### Strengths and limitations

Our review has both strengths and limitations. We comprehensively searched a very large number of abstracts, and data from included studies were double-checked. We were able to include studies in English, French, and Spanish, from all years of publication. However, it is possible that some studies which would have met our inclusion criteria were missed and we did not search the grey literature for our first two aims. There were only four studies which met our inclusion criteria, which explored different methods, so we are not able to draw clear conclusions about the methods and modes of measurement that are best suited to support increased disclosures. Several aspects of quality, ethical and safety considerations were not well reported in any study, which limits our ability to comment on these very important aspects of measuring violence against children. However, we are able to draw on a larger pool of studies conducted in children and young people, but without a focus on SRGBV, to propose avenues for future research in this area. Our search strategy and inclusion criteria was for studies which compared different modes of administration. It is also possible that different locations may yield different levels of disclosure e.g. asking about SRGBV in schools, where perpetrators are likely to be present, versus outside of schools, where perpetrators may be absent, and we did not seek to assess this in our review. Sex and type of violence experienced may also affect disclosure but there are insufficient studies to examine this. Finally, we purposively selected multi country surveys based on a narrative review of the literature and a scan of institutional websites. Our selection of surveys is therefore not representative or exhaustive, but serves to illustrate some key, and widely used, approaches to measuring SRGBV in large scale surveys.

### Implications

There is a clear need for a research agenda to establish which methods support children and young people to disclose their experience of violence, with attention to types of violence as well as victimisation and perpetration. The prevalence of violence disclosed varies enormously by data collection method, to such an extent that is likely to make a large difference in understanding the health and prevention needs of children. For SRGBV in particular, several promising strategies for further testing emerge. Our review points to the importance of further testing the sealed envelope method alongside other anonymous methods – ACASI, web surveys, and an automated telephone data collection (ATDC) system. There may also be methods that augment interviewer-administered methods, such as computer-assisted personal interviewing **(**CAPI) or adding drawing to face-to-face interviews, which may support disclosures from younger children. There are also several methods which have been tested in adults not children. These include: ACASI with varying levels of anonymity and confidential conditions; [55–57] a timeline follow back retrospective reporting method, [58] anonymised envelope or postcard, [59] and a double list experiment. [60] It may be possible to adapt and use these methods for some age groups of children.

It is important that further research examines both mode of administration as well as location of data collection while noting that other factors – age, sex, context of violence – could also affect disclosure as sensitivity of questions may differ by these factors. While self-completion methods may result in higher disclosure, administering them in schools where classrooms may be crowded is challenging logistically and ethically. Disclosure of violence may also be affected by who is present in the room, the training of researchers, and how the research is perceived in the school and community. Experimental or quasi-experimental designs should be used to ensure that only one variable e.g. mode of administration, location of data collection, differs at a time. Efforts to describe the logistics, costs, benefits and challenges of each data collection method tested will enable other researchers to make decisions about their method choice. Such research should also endeavour to seek feedback from young people on data collection and assess safety and child protection considerations. Efforts to understand how each data collection method is received and perceived by children and young people will require additional questions, or feedback interviews. Data generated from these efforts will allow researchers to improve the safety of these methods, address concerns young people raise, and ensure children’s voices are included in the design and testing of these methods.

Our findings imply that the methodology in current use in international surveys in LMICs may not be the best placed to support children’s disclosures of SRGBV. The VACS and DHS use face-to-face interviews in households, which may result in lower levels of disclosures versus fully anonymous methods. All routine school-based surveys (GSHS, HBSC, TIMSS & PIRLS) use self-completion questionnaires, with responses provided on paper or computer/tablet depending on the survey. [17–19, 21] The only survey testing written self-report for SRGBV suggests that this method may also produce lower levels of disclosure. [40] There is an urgent need to explore methodologies to best support children’s disclosures in these large-scale data collection methods, particularly since several large scale surveys are not conducted with teachers present.

It is likely that face-to-face interviewing will remain popular since it is easy to implement, allows longer questionnaires, can overcome literacy challenges due to age and education and the interviewer can ensure privacy of interview and support to participants. Interviewer training is likely to be very important in increasing disclosures since evidence from healthcare settings suggests that face-to-face reports to trusted individuals may be higher than self-complete options. [42, 61].

With respect to anonymous methods, these have strengths and limitations. For example, sealed envelopes are only practical to administer in relation to a very small number of questions due to the time needed to complete them. They can however be used with a wide age range of children, and could be used to ask the most sensitive questions which may be affected more by mode of administration. CASI would be advantageous in a setting with good literacy, but is of limited utility with younger children or in low literacy settings. ACASI can support participation in low literacy settings but takes longer to complete and so requires shorter questionnaire length. None of the studies we found assessing disclosure of violence reported on ease of use of ACASI. However, in one study on sexual behaviour, adult participants reported that ACASI was easy to use and private. [62] In another study, which evaluated the use of ACASI with adolescent girls in eastern Democratic Republic of Congo and refugee camps along the Sudan-Ethiopia border, the majority of girls found ACASI easy to use. In this study, level of education, rather than age, was associated with survey understanding. [63].

## Conclusions

There is a limited body of evidence on method of data collection and its impact on disclosure of SRGBV. Further research is needed to investigate how to best support children of younger and older ages, boys and girls, and other groups to fully disclose their experiences. Research is needed to further examine the influence of method of data collection, as well as other elements of data collection, in order to ensure that data collected on SRGBV is sufficiently accurate to inform intervention and service design and delivery.

## Data Availability

The datasets generated and/or analysed during the current study are not publicly available as the raw datasets are too big, but are available from the corresponding author on reasonable request.

## Abbreviations

ACASI: Audio computer-assisted self-interview
ATDC: Automated telephone data collection
CAPI: Computer-assisted personal interviewing
CASI: Computer-assisted self-interview
DHS: Demographic and Health Surveys
FTFI: Face-to-face interview
GSHS: Global School Health Surveys
HBSC: Health Behaviour in School-Aged Children Surveys
IPV: Intimate partner violence
LMICs: Low- and middle-income countries
PIRLS: Progress in International Reading Literacy Study
SRGBV: School-related gender-based violence
SDGs: Sustainable Development Goals
TIMSS: Trends in International Mathematics and Science Study
VACS: Violence Against Children and Youth Surveys
